# Sleep quality during and after severe acute respiratory syndrome coronavirus 2 (COVID‐19) lockdowns in the UK: Results from the SleepQuest study

**DOI:** 10.1111/jsr.14205

**Published:** 2024-04-23

**Authors:** Jonathan Blackman, Victoria Grace Gabb, Neil Carrigan, Alfie Wearn, Saba Meky, James Selwood, Bhavisha Desai, Hugh D. Piggins, Nicholas Turner, Rosemary Greenwood, Elizabeth Coulthard

**Affiliations:** ^1^ Institute of Clinical Neurosciences University of Bristol Bristol UK; ^2^ Bristol Brain Centre Southmead Hospital, North Bristol NHS Trust Bristol UK; ^3^ NIHR Bristol Biomedical Research Centre University of Bristol Bristol UK; ^4^ Department of Psychology University of Bath Bath UK; ^5^ School of Physiology, Pharmacology and Neuroscience University of Bristol Bristol UK; ^6^ Population Health Sciences Institute Bristol Medical School, University of Bristol Bristol UK; ^7^ NIHR Research & Design Service South West University Hospitals Bristol and Weston NHS Foundation Trust, Education & Research Centre Bristol UK

**Keywords:** coronavirus, COVID‐19, mental health, older adults, pandemic, sleep

## Abstract

Sleep is fundamental to health. The aim of this study was to analyse and determine factors predicting sleep quality during and after national lockdowns due to severe acute respiratory syndrome coronavirus 2 (COVID‐19) in the UK. A longitudinal online survey‐based study (SleepQuest) involving UK adults was administered in Spring 2020, Winter 2020, and Winter 2022 including questionnaires probing sleep quality, depression, anxiety, beliefs about sleep, demographics, COVID‐19 status, and exercise. The primary outcome was sleep quality (Pittsburgh Sleep Quality Index). A linear mixed‐effects model evaluated factors associated with baseline and longitudinal sleep quality. Complete data were provided by 3306 participants in Spring 2020, 2196 participants in Winter 2020, and 1193 in Winter 2022. Participants were mostly female (73.8%), white (97.4%), and aged over 50 years (81.0%). On average, participants reported poor sleep quality in Spring 2020 (mean [SD] Pittsburgh Sleep Quality Index score = 6.59 [3.6]) and Winter 2020 (mean [SD] Pittsburgh Sleep Quality Index score = 6.44 [3.6]), with improved but still poor sleep quality in Winter 2022 (mean [SD] Pittsburgh Sleep Quality Index score = 6.17 [3.5]). Improved sleep quality was driven by better subjective sleep and reduced daytime dysfunction and sleep latency. Being female, older, having caring responsibilities, working nightshifts, and reporting higher levels of depression, anxiety, and unhelpful beliefs about sleep were associated with worse baseline PSQI scores. Better sleep quality was associated with more days exercising per week at baseline. Interventions focusing on improving mental health, exercise, and attitudes towards sleep, particularly in at‐risk groups, may improve sleep‐related outcomes in future pandemics.

## 1 INTRODUCTION

The novel coronavirus 2019‐nCov/SARS‐Cov‐2 (COVID‐19) outbreak beginning in late 2019 motivated various policy responses from governments across the world, from restrictions on who could leave their homes and for what reasons (“lockdowns”) to vaccination rollouts and economic support. Restrictions in the UK, which included school and workplace closures, stay‐at‐home requirements, restrictions on public events and transport, and “shielding” (or self‐isolation) of at‐risk groups, have been considered among the most stringent (Hale et al., 2023). The COVID‐19 pandemic and its associated “containment and closure” policies placed a substantial psychological and physical burden on the population of all affected countries (Lopez‐Leon et al., 2021; Rogers et al., 2020), including a likely adverse impact on sleep (Jahrami et al., 2021; Souza et al., 2021).

Sleep is a biological need and essential to good health (Ramar et al., 2021), influencing cardiovascular (Korostovtseva et al., 2021), metabolic (Farr & Mantzoros, 2018) and mental health (Scott et al., 2021a) outcomes. Sleep is also fundamental to brain health. Sleep disturbances are a risk factor for neurodegenerative diseases including Alzheimer's disease (Kuang et al., 2021). Sleep and immunity are bidirectionally linked: poor sleep is both a symptom of and a risk factor for contracting respiratory infections and other diseases (Garbarino et al., 2021; Jones et al., 2022). Both acute COVID‐19 infection and long‐COVID are associated with sleep disturbances and fatigue (Lasselin et al., 2019; Merikanto et al., 2023). Restrictions to movement, daylight exposure, socialising, and daily activities, combined with fears about safety and wellbeing during the pandemic, may have also impacted sleep through stress, depression, anxiety, disruption to circadian rhythms and sleep pressure, and worse sleep hygiene (Salehinejad et al., 2022).

In this study, we determined whether sleep quality changed between the first and second lockdowns and after restrictions were lifted in the UK, and whether certain demographics, mental health, or lifestyle factors influenced sleep quality. We were particularly interested in whether older adults were at an increased risk of poor sleep quality during the COVID‐19 pandemic. Few studies have focussed on older adults; however, they are one of the most at‐risk groups for sleep deprivation and COVID‐19 (Jahrami et al., 2021). Older adults are at an increased risk of serious complications and mortality resulting from COVID‐19 (Bartleson et al., 2021; Ho et al., 2020), may have poorer sleep that could influence COVID‐19 outcomes (Pires et al., 2021a), and were disproportionately affected by the closure and containment policies seen in the UK (e.g. shielding), meaning older adults may have also experienced reduced exposure to both sunlight and social zeitgebers to align their circadian rhythms (Pires et al., 2021b).

### 1.1 The SleepQuest study

SleepQuest employs a longitudinal study design, allowing unique comparison of data from the same individuals during two periods of lockdown in the UK and after nearly all restrictions were lifted within the UK. Participants were invited to complete an online questionnaire three times, utilising a uniquely large sample size to address two main aims:

1. To describe sleep quality at the onset of the COVID‐19 pandemic, and compare this with sleep quality 6 months later and after “return to normal”.
2. To determine factors predicting baseline and change in sleep quality over time.

## 2 METHODS

### 2.1 Participants and study design

SleepQuest was a longitudinal web‐based survey hosted on the REDCap system at the University of Bristol, UK, open to all UK adults over the age of 18 years. Data were collected at two timepoints during periods of UK national lockdown (T1: 29/04/2020–13/05/2020; T2: 05/11/2020–02/12/2020), and a third timepoint that followed relaxation of all lockdown rules (T3: 05/12/2022–19/12/2022). Participants were provided with information online before consenting to take part. The questionnaire took approximately 20 min to complete at each timepoint.

**FIGURE 1:**
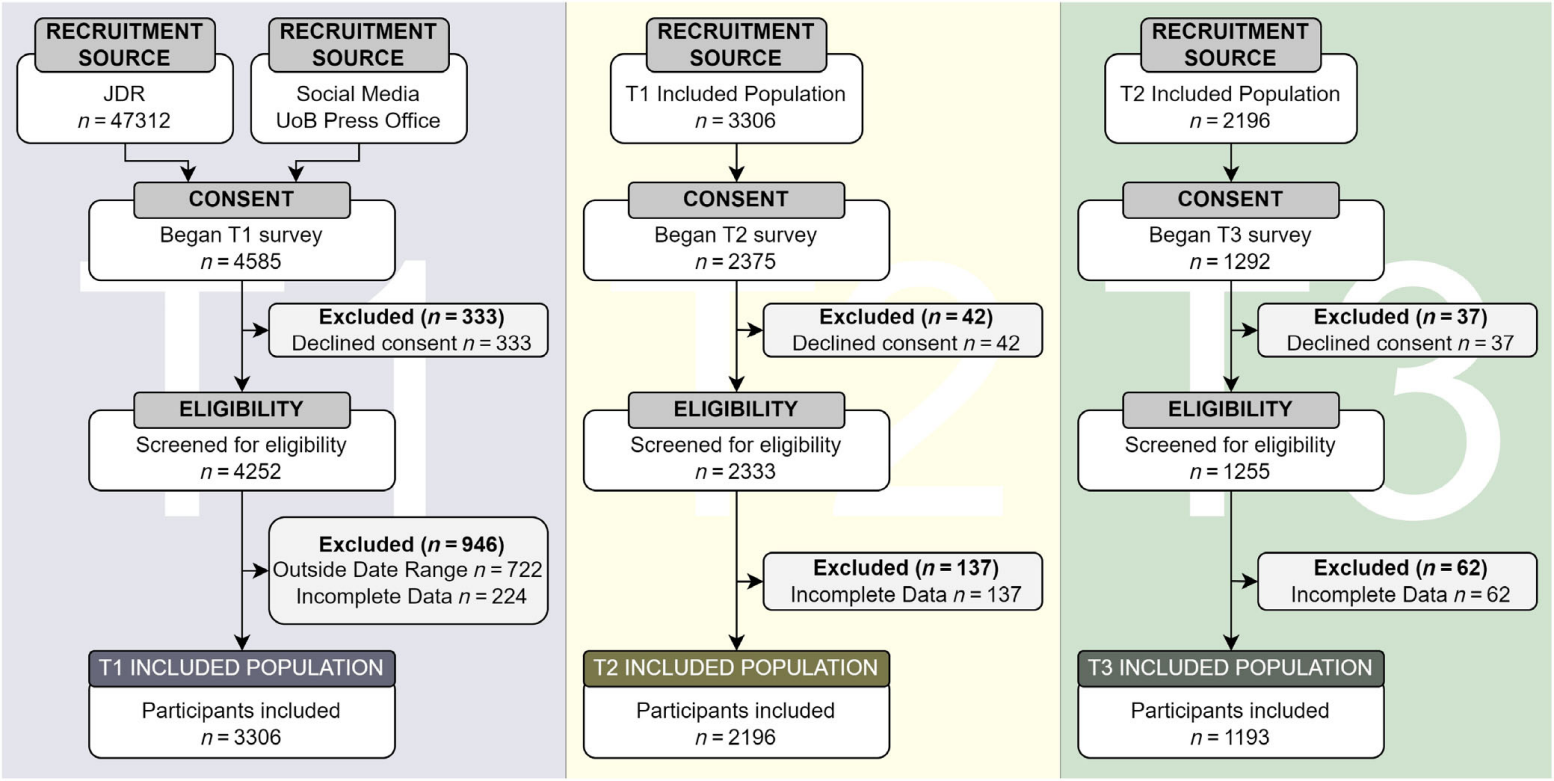
SleepQuest participation selection flowchart. Flowchart detailing participation selection during lockdown periods Timepoints represented by T1 (blue), T2 (yellow) and T3 (green).

To prioritise recruitment of older adults, participants were primarily recruited via the Join Dementia Research (JDR) database managed by the National Institute for Health Research in collaboration with the charitable organisations Alzheimer's Society, Alzheimer's Research UK, and Alzheimer's Scotland. The register comprises over 45,000 people who have agreed to be contacted about research opportunities. While JDR targets recruitment of people with dementia and caregivers, this is not mandatory, and many volunteers are healthy older adults without diagnoses or caring responsibilities. Email invitations were sent to JDR participants summarising the SleepQuest study with an opportunity to enrol via a website link. Participants were invited for follow‐up via direct email. To encourage recruitment of younger and middle‐aged adults to explore the association between age and sleep quality, the study was also advertised via Twitter and Facebook, through press releases by the University of Bristol public relations office, and through personal social media accounts. For full details of participant selection, see Figure 1.

Ethical approval for the study was through the Faculty of Health Sciences Research Ethics Committee, University of Bristol (FREC ref: 103244).

### 2.2 Measures

#### 2.2.1 Sociodemographic information

General sociodemographic information was collected including age, gender, ethnicity, education, employment (including night‐shift work), changes to employment due to COVID‐19, number of dependents, caring responsibilities, smoking and alcohol consumption, medical comorbidities (including presence of sleep disorders or dementia), and presence of confirmed or suspected COVID‐19 infection.

#### 2.2.2 Sleep quality

The Pittsburgh Sleep Quality Index (PSQI) is a self‐report, validated questionnaire designed to assess multiple aspects of sleep over the previous month (Buysse et al., 1989). A total of 19 individual items generate seven component scores whose summation provides a global sleep quality score. Component scores cover subjective sleep quality, sleep latency, sleep duration, habitual sleep efficiency, sleep disturbances, use of sleep medication, and daytime dysfunction. Scoring is based on a four‐point scale ranging from 0 to 3, with increased values representing more compromised sleep. A global score of 5 or greater indicates a “poor” sleeper (Buysse et al., 1989). The PSQI has good internal consistency and reliability (Cronbach's *α* = 0.83) for its seven components (Buysse et al., 1989).

#### 2.2.3 The Patient Health Questionnaire (PHQ‐8)

The PHQ‐8 (Kroenke et al., 2009) is an eight‐item self‐report measure assessing depressive symptoms. It removes the ninth item from the more widely used PHQ‐9 (“thoughts that you would be better off dead or of hurting yourself in some way”), as recommended in self‐administered internet surveys where further probing about positive responses to item 9 are not possible (Kroenke & Spitzer, 2002). Scores range from 0 to 24, with scores equal or greater than 10 indicating depression may be present. It has been shown to shown to be a valid diagnostic and severity measure in large clinical studies (Kroenke & Spitzer, 2002).

#### 2.2.4 The Generalised Anxiety Disorder Questionnaire (GAD‐7)

The GAD‐7 is a validated self‐report questionnaire that assesses generalised anxiety using seven items with four‐point Likert scales. Total scores range from 0 to 21, with clinical cut‐offs of 5, 10 and 15 for mild, moderate and severe anxiety, respectively (Spitzer et al., 2006).

#### 2.2.5 Dysfunctional Attitudes and Beliefs About Sleep (DBAS‐16)

The DBAS‐16 is a 16‐item self‐report measure assessing beliefs and attitudes towards sleep using a Likert‐type scale ranging from 0 to 10 for items such as “I am worried that I may lose control over my abilities to sleep”. Higher scores represent potentially unhelpful attitudes around sleep. It has been shown to be a reliable and valid measure of sleep‐related cognition in clinical samples (Morin et al., 2007).

#### 2.2.6 Exercise and daylight exposure

We captured the number of days in the previous week that any time was spent outside and the mean duration of these occasions. The number of days in the preceding week in which the participant had engaged in moderate to vigorous exercise (defined as activity that raises heart rate, increases breathing rate and makes them feel warmer) for ≥ 15 min was also captured.

### 2.3 Statistical analysis

R version 4.2.2 (2022‐10‐31) – “Innocent and Trusting” and RStudio (v2023.03.1 + 446) statistical software was used for data cleaning and analysis. Analysis of each timepoint included only participants supplying a full dataset for that timepoint. Post‐hoc sensitivity analyses were conducted to check whether findings were replicable when only including participants who completed all three timepoints. Descriptive statistics were used to characterise participants' demographics, health status, depression and anxiety, attitudes towards sleep, and time spent outside and exercising. Unpaired *t*‐tests assessed population‐level self‐reported change in sleep quality, depression, anxiety and dysfunctional sleep beliefs as reflected by total PSQI, PHQ‐8, GAD‐7 and DBAS‐16 scores, respectively, at timepoints T1 versus T2, T2 versus T3, and T1 versus T3. Population‐level change in each of the seven PSQI sub‐components was also compared using the Wilcoxon Rank Sum Test. A significance level of 0.05 was utilised.

Factors predicting change in sleep were assessed using linear mixed‐model analysis (LMM) using the *lme* function in R. PSQI total scores at timepoints T1, T2 and T3 were dependent variables for analysis. An intraclass correlation coefficient of 0.762 calculated from a grand mean (null) model suggested significant intra‐individual clustering, thus validating an LMM approach. Timepoint was assigned as 0, 1 and 5, respectively, for timepoints T1, T2 and T3, reflecting the proportional interval between data collection periods. Log‐likelihood values derived from unconditional models with time as a fixed effect (random intercept) and time as a random effect (random slope and intercept models) were compared by ANOVA with the latter providing improved model fit (*p* < 0.001) and therefore utilised for analysis. The final conditional model incorporated continuous covariates including age at baseline, PHQ‐8, GAD‐7 and DBAS‐16 total scores, days of exercise and daylight exposure per week, and dummy variables to incorporate gender, COVID‐19 infection within 6 months, nightshift work, presence of cognitive impairment/dementia, presence of children in the household, outdoor employment, and carer status. Covariates were selected by systematic examination of each collected data variable for its potential (hypothetically or evidentially) to influence the dependent variable (total PSQI). The final model specification therefore utilised participant identity as a random effect with interactions between covariates and timepoint included as a fixed effect of the form:

$$\begin{array}{l} \text{Total PSQI Score}\sim \text{Timepoint}+\text{Covariates}+\text{Timepoint}\times \text{Covariates}\\ \;+\;\left(\text{Timepoint}|\text{Participant}\right). \end{array}$$

The final adjusted regression model was checked for multicollinearity (Variance Inflation Factors < 10), normality of errors, homoskedasticity and linearity.

## 3 RESULTS

### 3.1 Cohort demographics

A total of 3306 people completed the minimum dataset during the first full lockdown (T1), mean (SD) age 59.1 (13.3) years, gender (F:M) = 0.75, 2391 people from the second lockdown (T2), mean (SD) age 60.6 (12.5) years, gender (F:M) = 0.84, and 1193 from the third timepoint (T3), mean (SD) age 61.5 (11.7) years, gender (F:M) = 0.87. Sensitivity analyses revealed no differences in the trends or their statistical significance when considering only participants who completed all three timepoints. Participant characteristics are provided in Table 1.

**TABLE 1:** Participant characteristics at timepoints T1, T2 and T3.

Demographic	T1	T2	T3
Participants, *n*	3306	2196	1193
Age (years), *mean (SD)*	59.1 (13.3)	60.6 (12.5)	61.5 (11.7)
Age category, *n (%)*			
18–29 years	152 (4.6)	84 (3.8)	38 (3.2)
30–49 years	505 (15.3)	266 (12.1)	119 (10)
> 50 years	2649 (80.1)	1846 (84.1)	1036 (86.8)
Gender, *n (%)*			
Female	2475 (74.9)	1623 (73.9)	841 (70.5)
Male	826 (25)	570 (26)	350 (29.3)
Other/prefer not to say	5 (0.2)	3 (0.1)	2 (0.2)
Ethnicity, *n (%)*			
Prefer not to say	4 (0.1)	2 (0.1)	2 (0.2)
Asian or Asian British	31 (0.9)	14 (0.6)	6 (0.5)
Black or Black British – Caribbean, African	8 (0.2)	2 (0.1)	0
Chinese or Chinese British	10 (0.3)	6 (0.3)	4 (0.3)
Middle Eastern or Middle Eastern British	5 (0.2)	1 (0)	0
Mixed Race – White & Black/Black British	4 (0.1)	3 (0.1)	0
Mixed Race – Other	29 (0.9)	19 (0.9)	9 (0.8)
White – British, Irish, other	3206 (97)	2144 (97.6)	1168 (97.9)
Other ethnicity	9 (0.3)	5 (0.2)	4 (0.3)
Dementia/cognitive impairment, *n (%)*	70 (2.1)	30 (1.4)	13 (1.1)
Caring responsibilities, *n (%)*	701 (21.2)	457 (20.8)	229 (19.2)
Recent (< 6 months) COVID‐19 Infection, *n (%)*	316 (9.6)	93 (4.2)	298 (25)
Diagnosed sleep disorder, *n (%)*	324 (9.8)	182 (8.3)	97 (8.1)

### 3.2 Baseline sleep quality during lockdown restrictions

Poor sleep quality was seen within this cohort. Mean (SD) PSQI score was 6.59 (3.6), higher than a large community sample of older adults (PSQI mean 5.4; Sun et al., 2020), and considerably higher on average than the cut‐off of ≥ 5 recognised as indicative of poor‐quality sleep (Buysse et al., 1989). Sub‐component analysis of the PSQI revealed sleep disturbance (mean = 1.39), sleep latency (mean = 1.21), subjective quality (mean = 1.16) and sleep efficiency (mean = 1.08) domains as principal contributors towards increased total PSQI score.

### 3.3 Overall sleep quality during and post‐lockdown periods

The mean total PSQI decreased (indicating better sleep) between the first and second lockdowns, reaching statistical significance (Mean Diff = −0.15; *p* = 0.015). The proportion of participants meeting the cut‐off of ≥ 5 fell from 54.8% at T1 to 52.0% at T2, to 50.2% at T3. Subcomponent analysis revealed a significant reduction in PSQI sleep disturbance score (Mean Diff = −0.07; *p* < 0.001), PSQI daytime dysfunction score (Mean Diff = −0.08; *p* < 0.001) and PSQI sleep latency score (Mean Diff = −0.04; *p* = 0.024) during this time (Table 2).

**TABLE 2:** Population level mean PSQI, PHQ‐8, GAD‐7 and DBAS scores at T1, T2 and T3.

Instrument	T1 Mean (SD)	T2 Mean (SD)	T3 Mean (SD)	*p*‐Value^a^		
				T1/T2	T1/T3	T2/T3
PSQI total	6.59 (3.6)	6.44 (3.6)	6.17 (3.5)	0.015^c^	< 0.001^c^	0.058^c^
Subjective sleep quality	1.16 (0.8)	1.16 (0.7)	1.11 (0.7)	0.684	0.009	0.028
Subjective sleep quality “Good” or “Fairly Good” (% of cohort)	69.42	70.40	74.35	0.455	0.002	0.016
Sleep latency	1.21 (1.0)	1.17 (1.0)	1.09 (0.9)	0.024	< 0.001	0.085
Sleep duration	0.53 (0.8)	0.55 (0.8)	0.54 (0.8)	0.400	0.843	0.644
Sleep duration (hr per night)	6.88 (1.2)	6.83 (1.2)	6.85 (1.2)	0.519	0.762	0.831
Sleep efficiency	1.08 (1.1)	1.09 (1.0)	1.11 (1.1)	0.908	0.363	0.437
Sleep efficiency (%)	79.26 (13.3)	79.06 (13.2)	78.76 (13.2)	0.936	0.164	0.168
Sleep disturbances	1.39 (0.5)	1.32 (0.5)	1.31 (0.5)	< 0.001	< 0.001	0.264
Use of sleep medication	0.28 (0.8)	0.29 (0.8)	0.28 (0.8)	0.735	0.774	0.993
Use of any sleep medications (% of cohort)	13.70	14.12	14.17	0.692^c^	0.727^c^	1^c^
Daytime dysfunction	0.94 (0.7)	0.86 (0.6)	0.74 (0.7)	< 0.001	< 0.001	< 0.001
PHQ‐8 total	5.39 (5.1)	4.63 (4.8)	3.99 (4.6)	< 0.001^c^	< 0.001^c^	< 0.001^c^
GAD‐7 total	4.00 (4.5)	3.42 (4.3)	2.82 (4.1)	< 0.001^c^	< 0.001^c^	< 0.001^c^
DBAS total	3.82 (1.6)	3.62 (1.6)	^b^	< 0.001^c^	^b^	^b^

Abbreviations: DBAS‐16, Dysfunctional Attitudes and Beliefs About Sleep; GAD‐7, Generalised Anxiety and Disorder Questionnaire; PHQ‐8, Patient Health Questionnaire; PSQI, Pittsburgh Sleep Quality Index.

^a^ Comparisons made utilising Wilcoxon Rank Sum Test unless otherwise stated.

^b^ DBAS data not collected at Timepoint 3.

^c^ Unpaired *t*‐test.

**FIGURE 2:**
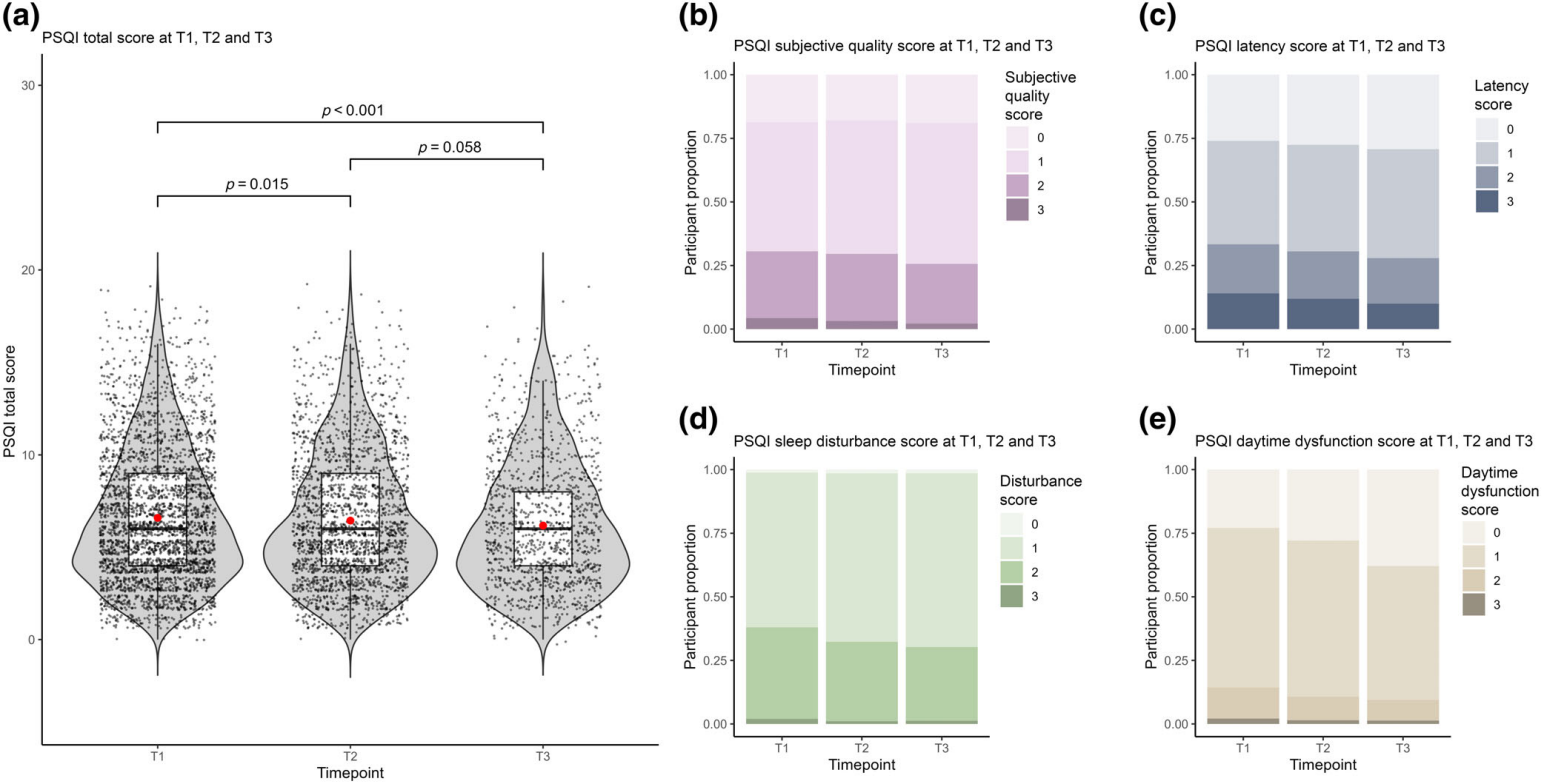
Mean population Pittsburgh Sleep Quality Index (PSQI) total and subcomponents by timepoint. Mean population‐level scores at timepoints T1, T2 and T3 for total PSQI score (a), and population scores for PSQI components reflecting subjective sleep quality (b), sleep latency (c), sleep disturbances (d) and daytime dysfunction (e).

Overall sleep quality was found to have improved post‐lockdown, with a reduced mean total PSQI score of 6.17 (SD = 3.5) at T3, a statistically significant improvement when compared with T1 (*p* < 0.001). The key contributory subcomponents accounting for this change from T1 to T3 were improvements in daytime dysfunction (Mean Diff = −0.20; *p* < 0.001), sleep latency (Mean Diff = −0.12; *p* < 0.001), subjective sleep quality (Mean Diff = −0.05; *p* = 0.009) and sleep disturbances (Mean Diff = −0.08; *p* < 0.001; Figure 2).

### 3.4 Population‐level changes in key factors known to influence sleep

**FIGURE 3:**
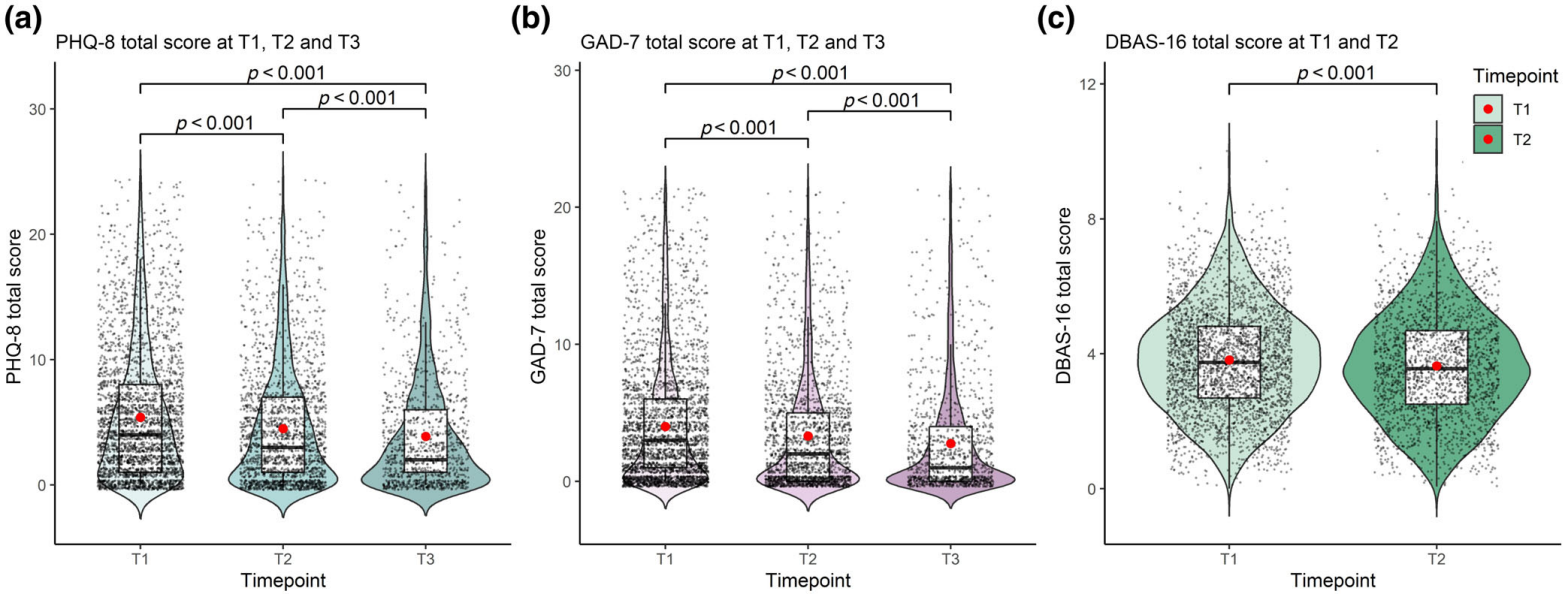
Mean Patient Health Questionnaire (PHQ‐8), Generalised Anxiety Disorder Questionnaire (GAD‐7) and Dysfunctional Attitudes and Beliefs About Sleep (DBAS‐16) scores by timepoint. Mean population‐level scores at timepoints T1, T2 and T3 for PHQ‐8 (a), GAD‐7 (b), and at timepoints T1 and T2 for DBAS‐16 (c).

Depression, anxiety and unhelpful attitudes towards sleep were measured by the use of PHQ‐8, GAD‐7 and DBAS‐16, respectively. There were statistically significant improvements in all three metrics between T1 and T2. This trend continued beyond the lockdown periods, with improvements from T1 to T3 seen in GAD‐7 (Mean Diff = −1.18, *p* < 0.001) and PHQ‐8 (Mean Diff = −1.40, *p* < 0.001; Figure 3; Table 2).

### 3.5 Factors predicting sleep quality during and after lockdown periods

A LMM was used to determine factors predicting sleep quality at baseline and those predicting a change in sleep quality over time. Female gender, increased age, caring responsibilities, higher levels of self‐reported depression, anxiety and the presence of unhelpful beliefs pertaining to sleep were all associated with a worse (higher) total PSQI score at baseline. The largest effect sizes were found in nightshift workers (Est = 1.460, *p* < 0.001), those with unhelpful beliefs pertaining to sleep (Est = 0.384, *p* < 0.001) and those with depressive symptoms (Est = 0.291, *p* < 0.001). More frequent exercise during the week was associated with improved sleep, though the effect size was relatively small (Est = −0.043, *p* = 0.008; Table 3). Trends were similar in the sensitivity analysis, though effect sizes were smaller, however, and the association between exercise and sleep was no longer statistically significant (*p* > 0.05).

**TABLE 3:** Factors predicting total PSQI.

Variable	*β* Coef.	Std. Error	*p*‐Value	Interaction	*β* Coef.	Std. Error	*p*‐Value
Time	0.105	0.10	0.300				
Female gender	0.580	0.11	< 0.001	Time × Female gender	−0.014	0.03	0.630
Age	0.039	0.00	< 0.001	Time × Age	0.000	0.00	0.720
Outside employment	−0.081	0.12	0.496	Time × Outside employment	0.020	0.04	0.616
Nightshift work	1.460	0.40	< 0.001	Time × Nightshift work	−0.305	0.11	0.008
Caring responsibilities	0.297	0.09	0.001	Time × Caring responsibilities	0.009	0.04	0.802
Cognitive impairment/dementia	−0.090	0.31	0.772	Time × Cognitive impairment/dementia	−0.036	0.11	0.742
Children	0.063	0.06	0.307	Time × Children	0.009	0.03	0.723
No. of days exercise	−0.043	0.02	0.008	Time × No. of days exercise	0.005	0.01	0.428
Days/week of daylight exposure	−0.004	0.00	0.219	Time × Days/week of daylight exposure	0.000	0.00	0.786
PHQ‐8 total	0.291	0.00	< 0.001	Time × PHQ‐8 total	0.014	0.00	0.004
GAD‐7 total	0.072	0.01	< 0.001	Time × GAD‐7 total	−0.011	0.01	0.034
DBAS‐16 total	0.384	0.03	< 0.001	Time × DBAS‐16 total	−0.023	0.01	0.010
COVID‐19 infection (< 6 months)	0.143	0.12	0.224	Time × COVID‐19 infection (< 6 months)	−0.028	0.04	0.436

*Note*: Fixed effects from mixed effects model with interaction terms including relationship with timepoint (T1, T2 and T3 set as 0, 1 and 5, respectively, to account for interval ratio in data collection). Random effects: Intercept Std. Dev = 1.63, Timepoint Std. Dev = 0.10.

Abbreviations: DBAS‐16, Dysfunctional Attitudes and Beliefs About Sleep; GAD‐7, Generalised Anxiety Disorder Questionnaire; PHQ‐8, Patient Health Questionnaire.

**FIGURE 4:**
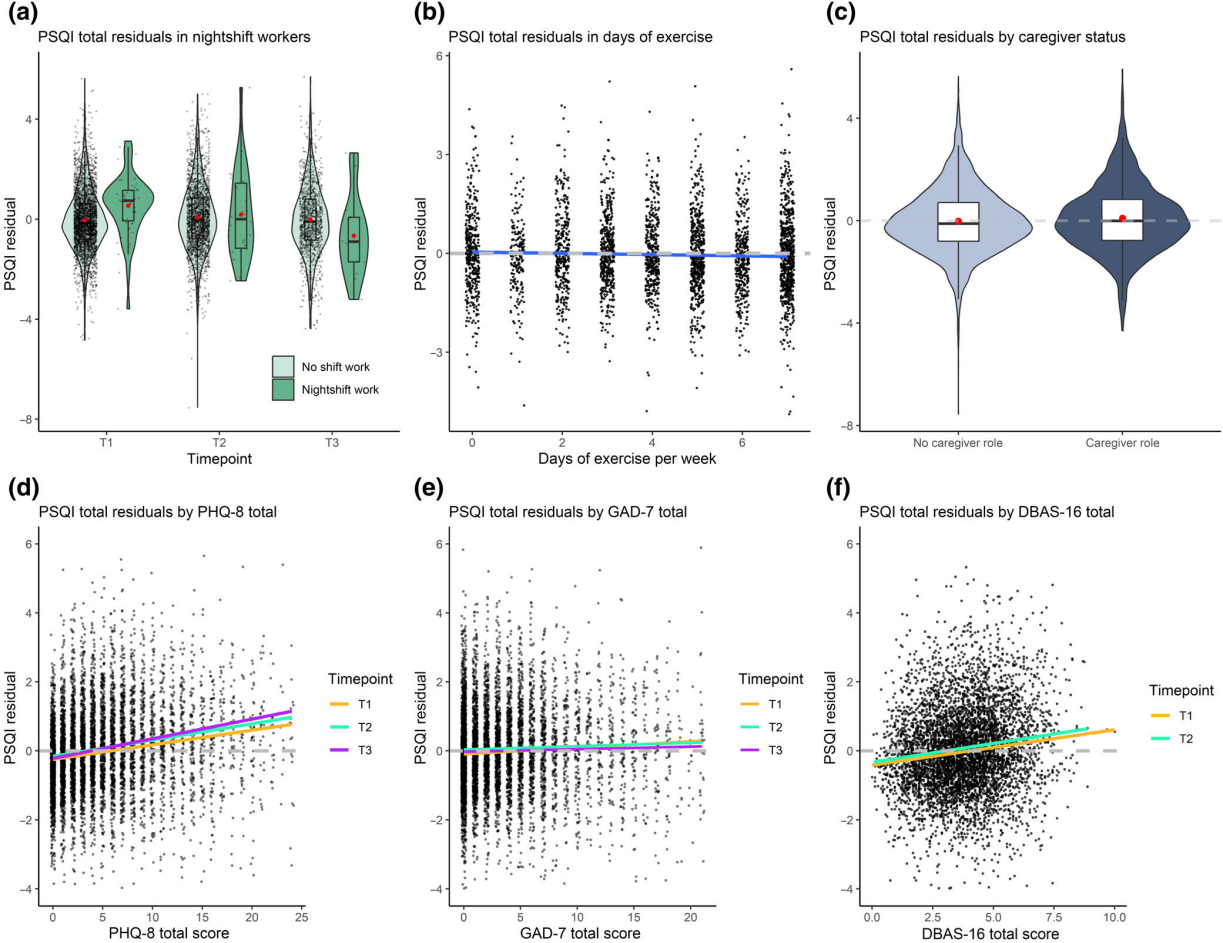
Key associations with Pittsburgh Sleep Quality Index (PSQI) Score from linear mixed‐model (LMM). Key variables found to be associated with PSQI total and their residual values from linear mixed effect modelling controlling for all other variables* within the fully adjusted model. (a) The effect of nightshift work (dark green) demonstrates an adverse association at T1, which is attenuated at T2 and reversed at T3. (b) Number of days exercise is plotted against PSQI residuals controlling for all other variables. A linear best‐fit line (blue) crosses the intercept (grey) demonstrating the association with improved sleep at higher frequencies of exercise. (c) The positive association with PSQI total (i.e. worse quality sleep) of caregiver status (dark blue) against its absence (light blue) controlling for all other variables. (d) Depression as defined by Patient Health Questionnaire (PHQ‐8) total score plotted against PSQI residuals showing the relative stronger association at T3 (magenta) than at T1 (orange). (e) Anxiety as defined by Generalised Anxiety Disorder Questionnaire (GAD‐7) total score plotted against PSQI residuals showing the relative stronger association at T1 (orange) and T2 (cyan) than at T3 (magenta). (f) The positive association at T1 and T2 between unhelpful beliefs towards sleep as defined by Dysfunctional Attitudes and Beliefs About Sleep (DBAS‐16) and PSQI total.

The marked adverse effect of nightshift work seen at T1 was attenuated at T2 and reversed at T3 as demonstrated by its interaction with time (Est = −0.305, *p* = 0.008). Those with higher levels of depression reflected by PHQ‐8 trended towards a gradual worsening of sleep by T3 (Est = 0.014, *p* = 0.004), whilst those with increased anxiety and unhelpful beliefs regarding sleep trended towards gradual improvement of sleep by T3 (GAD‐7: Est = −0.011, *p* = 0.034; DBAS‐16: Est = −0.023, *p* = 0.010). Although effect sizes are small, this suggests that anxiety and unhelpful beliefs towards sleep were more strongly associated with poor‐quality sleep during the lockdown periods, with depression playing a greater role post‐pandemic (Figure 4; Table 3).

## 4 DISCUSSION

The rapid transmission of COVID‐19 in the UK prompted urgent and restrictive containment and closure measures to slow and reduce transmission of the virus in 2020 and 2021. This cohort study captured sleep quality and mental health data during three distinct COVID‐19 periods: spring 2020 (during the first national UK lockdown); winter 2020 (during the second national UK lockdown); and winter 2022 (about 8 months after all COVID‐19 restrictions were lifted in the UK).

### 4.1 Sleep quality during and after the COVID‐19 pandemic in the UK

Our analysis suggested that poor sleep quality was common in adults living in the UK during national lockdowns in 2020. We observed a small improvement in sleep quality following a “return to normal” in 2022 from 2020. Sleep disturbances were greatest early in the pandemic and improved through the pandemic, whilst daytime dysfunction and sleep latency were worse during both pandemic timepoints and improved 2 years on. At all timepoints, on average participants reported poor overall sleep quality (≥ 5 on total PSQI score) and poor sleep efficiency (≤ 85%) and a shorter sleep duration than typically recommended for adults (< 7 hours). Poor sleep quality in our sample may be partly explained by a high proportion of both older adults and females in our sample, as both were associated with worse subjective sleep quality. Several studies have identified increased vulnerability to poor sleep in older adults (Kim et al., 2021; Landry et al., 2015) and women (Jane et al., 2022; Salfi et al., 2020; Salfi et al., 2023) during the COVID‐19 pandemic, and our study suggests this trend continued after restrictions were lifted and infection rates decreased.

Towards the start of the pandemic, several participant characteristics predicted worse sleep quality. In addition to older age and female gender, having caring responsibilities, working nightshifts, and reporting higher levels of depression, anxiety and unhelpful attitudes towards sleep all predicted worse sleep quality. Poor sleep quality was highest in nightshift workers, those with unhelpful beliefs pertaining to sleep, and those with more depressive symptoms, suggesting groups who might most benefit from interventions aimed at improving sleep quality. People who fall into more than one of these at‐risk categories (e.g. female caregivers; Byun et al., 2016) may be at higher risk of poor sleep and its associated health complications.

Those who exercised more frequently during the week were less likely to report poor sleep quality, indicating a possible protective effect of regular exercise, although direction of causation cannot be established from our data and effect sizes were small. Spending time outdoors, having cognitive impairment or dementia, or working outside of the home were not associated with self‐reported sleep quality. Further research should aim to identify additional protective factors for sleep quality as targets for intervention.

### 4.2 Factors predicting change in sleep quality over time

Working nightshifts during the pandemic was associated with poor sleep at the start of the pandemic, with slight improvement seen later in the pandemic, and conversely better sleep quality at the last timepoint. It is likely that people working nightshifts during the pandemic were “key workers”, so poor sleep quality early in the pandemic may have been influenced by greater exposure to COVID‐19 (Topriceanu et al., 2021), and likelihood of experiencing stress, anxiety, or depression (Bu et al., 2022). Our findings are in line with cross‐sectional studies that have identified poorer sleep quality in healthcare workers and the police working more nightshifts (James et al., 2023; Omar et al., 2022; Power et al., 2022). Our study suggests that during health crises, employers of frontline workers should take additional steps to support their employees who are more likely to be sleep‐deprived, as poor sleep quality is associated with increased risk of health complications and workplace and road traffic accidents (Chattu et al., 2018).

Overall, our sample had low levels of anxiety and depression, even at early stages of the pandemic, with improvements in anxiety and depression observed over time. One possible explanation is that some of the observed anxiety and depressive symptoms at the start of the pandemic were a transient reaction to the pandemic itself to which people adapted to over time or was alleviated during periods of less intense lockdowns (Bendau et al., 2021; Lindner et al., 2022). Interestingly, heightened anxiety and unhelpful beliefs towards sleep were more strongly associated with poor sleep quality during the lockdown periods, whilst depression was more strongly associated with poor post‐pandemic sleep quality. Although sleep quality was worse in those with mental health problems, our study cannot determine whether poor sleep was a cause or consequence of mood disturbances. Sleep and mood have bidirectional relationships, whereby poor sleep can worsen mood, whilst sleep disturbances are a common symptom of mood disorders such as anxiety and depression (Alvaro et al., 2013).

At‐risk groups (people with anxiety and depression, carers, nightshift workers, females and older adults) may benefit most from interventions or public health campaigns to improve sleep in future pandemics or epidemics. Our study suggests that encouraging regular exercise, treating anxiety and depression, and promoting positive attitudes towards sleep may improve sleep quality. Interventions such as cognitive‐behavioural therapy or exercise, which can target sleep and mood simultaneously (Lederman et al., 2019; Scott et al., 2021b), could be particularly beneficial in at‐risk groups.

### 4.3 Limitations

We captured data at three unique timepoints during the COVID‐19 pandemic. However, COVID‐19 infection rates, guidance and restrictions within the UK were dynamic, and changes in policy were often implemented at short notice. There was also regional variation in lockdown restrictions within the UK. Additional timepoints and bespoke questions about local rates and restrictions and which restrictions and recommendations participants were following at each timepoint, what their sleep quality was like before the pandemic, and factors felt to most impact sleep may have helped us to better understand and contextualise our results. Though not validated tools, these questions may have provided insights into behaviours or factors underlying our key findings, such as reasons behind the reduced sleep efficiency seen during the pandemic, but would also have increased participant burden.

Although the focus of the present study was on the impact of COVID‐19 lockdowns rather than acute infection, we asked people about COVID‐19 status in the 6 months prior to each timepoint, and did not identify an association between having a probable COVID‐19 infection in the previous 6 months and sleep quality. Patients with an active COVID‐19 infection are very likely to report sleep disturbances (Jahrami et al., 2021), and sleep disturbance and fatigue are considered symptoms of long‐COVID‐19 and are associated with more severe COVID‐19 infection (Merikanto et al., 2023). We anticipate that our findings may be influenced by a high proportion of cases being asymptomatic and not triggering testing, not limiting “recent infection” to a smaller timeframe (e.g. 2 weeks), limited availability of COVID‐19 testing kits during the follow‐up periods, and cessation of recommended testing at the final timepoint. Future studies could provide home‐testing kits or encourage reporting of relevant test results to complement analyses and help to disentangle the impact of the acute infection and the societal, psychological and lifestyle changes imposed by lockdowns.

Another limitation is the reliance on self‐reported sleep quality reported via the PSQI. The PSQI is the most commonly used measure of subjective sleep quality (Fabbri et al., 2021). However, subjective and objective measures of sleep quality often do not agree and capture different components of sleep, and this sleep discrepancy may be enhanced in those with sleep problems (Van Den Berg et al., 2008). Multidimensional approaches to sleep assessment would be preferable, utilising self‐report alongside wearable devices such as actigraphy or home‐based encephalography for deeper sleep profiling and screening of undiagnosed sleep disorders. Though we found statistically significant changes over time, it is also not well‐established what a minimal clinically important difference (MCID) is in the PSQI, and therefore it is difficult to conclude whether the changes observed represent meaningful change for participants. Research into MCID for different sleep measures would be beneficial.

Finally, restricting responses to online surveys may have created unintentional bias, and we had a disproportionate number of women, older adults and White participants. Therefore, recruitment bias restricts the generalisability of these results to the entire UK population. However, utilising online self‐report enabled us to recruit and retain a large cohort of participants in a short timeframe without risk to participants or the research team.

## 5 CONCLUSION

Since 2020, COVID‐19 has significantly impacted the world and global health. Further lockdowns due to COVID‐19 or other epidemics or pandemics are possible (Mariani et al., 2021). Characterising what sleep quality is like during a pandemic, and identifying at‐risk populations and potentially modifiable risk factors that might impact sleep are critical to preserve long‐term mental and physical health in the future.

To the best of our knowledge, this study is the first to longitudinally examine the long‐term trajectory of sleep quality, depression, and anxiety in the UK during and 2 years after the pandemic focussing on older adults. Our study demonstrates that though there have been significant improvements in subjective sleep quality since the pandemic, these improvements were relatively small. We should consider how to improve sleep quality, particularly in those most at‐risk, including nightshift workers, older adults, females and those with caring responsibilities, anxiety or depression – both in future pandemics and to preserve health and wellbeing during “normal times”.

## AUTHOR CONTRIBUTIONS

**Jonathan Blackman:** Formal analysis; writing – review and editing; writing – original draft; project administration; methodology. **Victoria Grace Gabb:** Formal analysis; writing – original draft; writing – review and editing; project administration; methodology. **Neil Carrigan:** Conceptualization; formal analysis; writing – original draft; methodology; writing – review and editing; project administration. **Alfie Wearn:** Conceptualization; formal analysis; writing – original draft; writing – review and editing; methodology; project administration. **Saba Meky:** Writing – review and editing. **James Selwood:** Formal analysis. **Bhavisha Desai:** Formal analysis. **Hugh D. Piggins:** Writing – review and editing. **Nicholas Turner:** Formal analysis; writing – review and editing; supervision. **Rosemary Greenwood:** Writing – review and editing. **Elizabeth Coulthard:** Conceptualization; formal analysis; supervision; methodology.

## FUNDING INFORMATION

This work was funded by a Above & Beyond grant (ABL‐2019‐20‐01) and by Alzheimer's Research UK (ARUK‐NC2019‐BB). AW was funded by BRACE. JB receives funding from Alzheimer's Research UK (supported by the Margaret Jost Fellowship and the Don Thoburn Memorial Scholarship) and the David Telling Charitable Trust. EC has received funding from BRACE and ARUK (Bristol & Bath Network). VG has received funding from Above & Beyond and NIHR Bristol Biomedical Research Centre.

## CONFLICT OF INTEREST STATEMENT

The authors declare that they have no competing interests.

## Data Availability

The data that support the findings of this study are openly available in Dementias Platform UK Data Portal at https://doi.org/10.48532/043000.
